# Coherence of the Surface EMG and Common Synaptic Input to Motor Neurons

**DOI:** 10.3389/fnhum.2018.00207

**Published:** 2018-06-11

**Authors:** Jakob L. Dideriksen, Francesco Negro, Deborah Falla, Signe R. Kristensen, Natalie Mrachacz-Kersting, Dario Farina

**Affiliations:** ^1^Center for Sensory-Motor Interaction, Department of Health Science and Technology, Aalborg University, Aalborg, Denmark; ^2^Department of Clinical and Experimental Sciences, Università degli Studi di Brescia, Brescia, Italy; ^3^Centre of Precision Rehabilitation for Spinal Pain, School of Sport, Exercise, and Rehabilitation Sciences, College of Life and Environmental Sciences, University of Birmingham, Birmingham, United Kingdom; ^4^Department of Bioengineering, Imperial College London, London, United Kingdom

**Keywords:** coherence, motor control, motor unit, surface EMG, synaptic input

## Abstract

Coherence between electromyographic (EMG) signals is often used to infer the common synaptic input to populations of motor neurons. This analysis, however, may be limited due to the filtering effect of the motor unit action potential waveforms. This study investigated the ability of surface EMG–EMG coherence to predict common synaptic input to motor neurons. Surface and intramuscular EMG were recorded from two locations of the tibialis anterior muscle during steady ankle dorsiflexions at 5 and 10% of the maximal force in 10 healthy individuals. The intramuscular EMG signals were decomposed to identify single motor unit spike trains. For each trial, the strength of the common input in different frequency bands was estimated from the coherence between two cumulative spike trains, generated from sets of single motor unit spike trains (reference measure). These coherence values were compared with those obtained from the coherence between the surface EMG signals (raw, rectified, and high-passed filtered at 250 Hz before rectification) using linear regression. Overall, the high-pass filtering of the EMG prior to rectification did not substantially change the results with respect to rectification only. For both signals, the correlation of EMG coherence with motor unit coherence was strong at 5% MVC (*r*^2^ > 0.8; *p* < 0.01), but only for frequencies > 5 Hz. At 10% MVC, the correlation between EMG and motor unit coherence was only significant for frequencies > 15 Hz (*r*^2^ > 0.8; *p* < 0.01). However, when using raw EMG for coherence analysis, the only significant relation with motor unit coherence was observed for the bandwidth 5–15 Hz (*r*^2^ > 0.68; *p* = 0.04). In all cases, there was no association between motor unit and EMG coherence for frequencies < 5 Hz (*r*^2^ ≤ 0.2; *p* ≥ 0.51). In addition, a substantial error in the best linear fit between motor unit and EMG coherence was always present. In conclusion, high-frequency (>5 Hz) common synaptic inputs to motor neurons can partly be estimated from the rectified surface EMG at low-level steady contractions. The results, however, suggest that this association is weakened with increasing contraction intensity and that input at lower frequencies during steady isometric contractions cannot be detected accurately by surface EMG coherence.

## Introduction

Coherence is a measure of the linear correlation between two signals that is frequently used in electrophysiology to investigate neural connectivity. Coherence is often applied to surface electromyographic (EMG) signals to investigate the connectivity of motor neuron pools. For example, EEG–EMG (corticomuscular) and EMG–EMG coherence have been used to estimate the spectral characteristics of the cortical input to motor neurons (Mima and Hallett, 1999; Lattari et al., 2010) or the common synaptic input to populations of motor neurons within and across muscles (Farmer et al., 2007; Keenan et al., 2012). In these analyses, specific emphasis is often given to the beta band (15–35 Hz; Salenius and Hari, 2003). The exact association between coherence measures of interference EMG or EEG signals and the correlation between synaptic inputs to motor neurons, however, are not fully known. For example, the potential benefits of rectification of the EMG signals have been extensively debated (Halliday and Farmer, 2010; Stegeman et al., 2010; Boonstra and Breakspear, 2012; Keenan et al., 2012). Recently, it was demonstrated that coherence analysis involving the raw EMG may underestimate correlation levels because the motor unit action potentials act as high-pass filters on the neural information, while, on the other hand, rectification of the EMG, which is a non-linear operation, imposes potential distortions of the signal (Farina et al., 2013; Negro et al., 2015). These findings suggest that several factors determine whether rectification is appropriate or not, such as the frequency band of interest and the force level. In addition, it has been suggested that high-pass filtering of the EMG prior to rectification increases EMG–EMG coherence (Boonstra and Breakspear, 2012), which may reflect a better characterization of the common synaptic input to the motor neurons.

Two recent studies directly addressed the association between coherence analysis of the EMG and the strength of correlation in synaptic input to motor neurons. Ward et al. (2013) observed that coherence values in the beta band using the rectified EMG predicted the values estimated from the correlation between pairs of motor unit spike trains better than when using the raw EMG (Ward et al., 2013). However, coherence between motor unit spike trains depends on the number of motor units used for the estimate and the use of pairs of units results in highly variable estimates (Farina et al., 2014; Farina and Negro, 2015). Dakin et al. (2014) electrically stimulated the vestibular system at frequencies in the range 1–20 Hz to provide a known cortical input to the gastrocnemius muscle and found significant coherence between the stimulation signal and the rectified EMG. However, the condition studied does not resemble voluntary muscle activation.

In this study, we analyzed intramuscular coherence concurrently from surface EMGs and motor unit spike trains detected from two regions of the tibialis anterior muscle during low force contraction. Coherence values obtained between cumulative spike trains (CSTs) comprising the spike trains of three motor units were used as reference estimates and were compared to the surface EMG–EMG coherence with rectification, without rectification, and with high-pass filtering prior to rectification. The aim was to systematically investigate the ability of surface EMG–EMG coherence to predict the common synaptic input to the motor neuron pool.

## Materials and Methods

### Experimental Procedure

Ten healthy volunteers (7 men, age: 24 ± 3 years) with no history of neurological conditions and free of medication participated in the study. All subjects provided written informed consent and all procedures were approved by the Scientific Ethics Committee of Northern Jutland, Denmark (reference number: N-20100067). The procedures were conducted according to the Declaration of Helsinki.

The subjects were seated comfortably in a chair with their hip and knee flexed at 90° and 130°, respectively. The right foot was strapped to a footplate that enabled measurement of the ankle dorsiflexion force. First, the maximum voluntary isometric contraction (MVC) dorsiflexion force was measured. The subject was asked to increase the force to the maximum over a period of 3 s and to maintain it for an additional 3 s with verbal encouragement. This procedure was repeated three times with breaks of 2 min in order to identify the highest force as the reference for defining relative submaximal forces.

**FIGURE 1:**
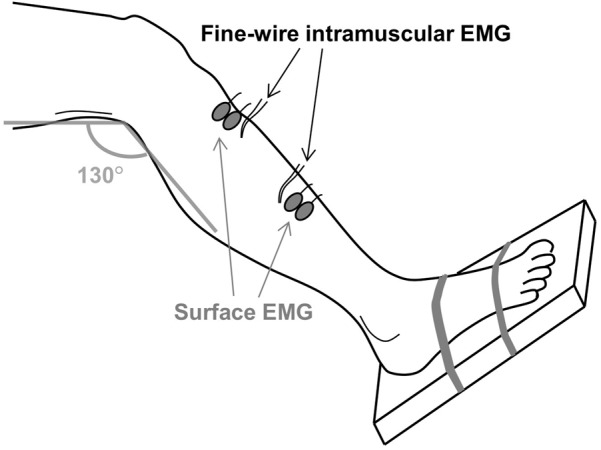
The experimental setup. Two pairs of fine-wire intramuscular EMG electrodes were inserted in the proximal and distal regions of the tibialis anterior muscle. Two pairs of surface EMG electrodes were attached near the two insertion sites. The foot was strapped to a footplate that enabled measurement of the ankle dorsiflexion force.

After the MVC, two pairs of bipolar surface and intramuscular EMG electrodes were positioned at the distal and proximal region of the tibialis anterior muscle, as illustrated in **Figure 1**. Inter-electrode distance for the pairs of surface electrodes was 2 cm. All electrodes were placed on the line between the tip of the fibula and the tip of the medial malleolus and the distance between the two pairs of surface EMG electrodes was maximized in order to minimize cross-talk. Prior to placement of the surface electrodes, the subject’s skin was prepared by gentle local abrasion using abrasive paste (Medic-Every, Parma, Italy) and cleansed with water. The areas selected for the insertion of intramuscular EMG electrodes were disinfected using alcohol swipes. Pairs of intramuscular EMG electrodes made of fine wire (50 μm diameter) Teflon-coated stainless steel (A-M Systems, Carlsborg, WA, United States) were inserted using a 23-gauge needle at a 45° angle to a depth of 5–10 mm. Each wire was cut to expose the cross section at the tip without insulation and 2–3 mm of the wires was bent backward. After insertion, the needle was removed leaving the wires in the muscle for the duration of the experiment. The quality of all signals was visually inspected. In case of poor signal quality, the corresponding channels were excluded from further analysis or the electrodes were inserted again. Both the surface and intramuscular EMG signals were sampled at 10 kHz and bandpass filtered from 10 Hz to 4.4 kHz using an analog EMG amplifier (OT Bioelettronica). Force was sampled at 10 kHz and low-pass filtered (cut-off frequency 5 Hz).

During the experiment, the subjects performed two isometric contractions of ankle dorsiflexions at 5% MVC and two at 10% MVC for 90 s each, in a random order. Force feedback was provided on a computer screen placed 2 m in front of the subject. A break of at least 2 min was given between each contraction.

### Data Analysis

**FIGURE 2:**
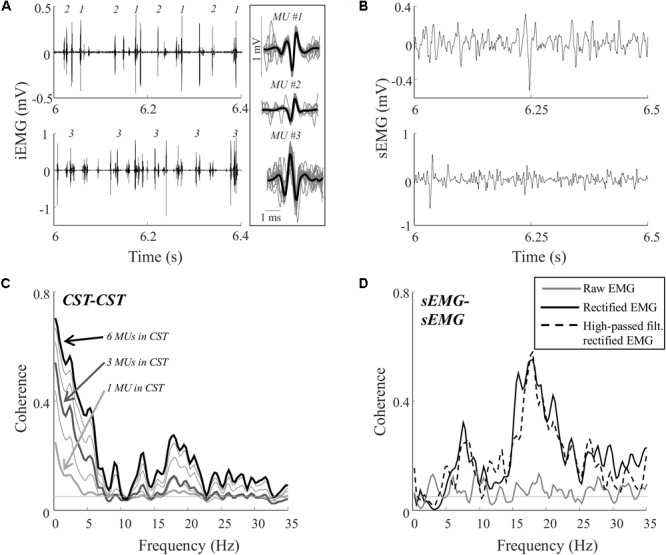
Representative data for one subject during a contraction at 10% MVC. Recordings of the proximal and distal intramuscular EMG electrodes **(A)** and the proximal and distal surface EMG electrodes **(B)**. From the two intramuscular EMG recordings, a total of 12 single motor unit spike trains were decomposed. The firing times of three of these motor units are represented by numbers in the intramuscular EMG traces **(A)**. In addition, the average action potential shape of these units is shown (averaged over all firings; black bold lines) superimposed on 25 of the action potentials (evenly distributed throughout the contraction; gray lines) from which the average potential was calculated. The total numbers of action potentials for the three units were 833, 892, and 994, respectively. **(C)** The coherence between CSTs consisting of different numbers of trains of motor unit action potentials. Each line represents the average of up to 100 random permutations. The coherence spectra obtained with 1, 3, and 6 motor units per CST are highlighted (bold lines). **(D)** The coherence between the two surface EMG recordings [raw, rectified, and/or high-pass filtered (>250 Hz) before rectification]. The coherence spectra were smoothed using a five-point hamming window. The thin, gray, dashed lines in **C,D** indicate the confidence limits.

Single motor unit spike trains were identified from the intramuscular EMG recordings using the algorithm described by (McGill et al., 2005; see **Figure 2** for examples). To avoid that the same motor unit detected by both intramuscular recordings was included in the analysis twice, the peak of the normalized cross-correlation function between pairs of spike trains across the two recording sites was calculated. If this value was higher than 30%, the spike train with fewest action potentials was excluded.

The signal-to-interference ratio (SIR) was calculated to estimate the degree to which the identified motor units contributed to the surface EMG signal. To compute SIR, the surface action potentials of the identified motor units were extracted by spike-triggered averaging and used to build a synthetic surface EMG signal made of the detected motor unit spike trains. SIR was then obtained as:

$$\begin{array}{c} SIR=(1-\frac{\bar{{\left(EMG-\Sigma_{i}(MUAP_{i}*ST_{i})\right)}^{2}}}{\bar{EMG^{2}}})\cdot 100\% \end{array}$$

where *EMG* is the raw surface EMG signal, *i* denotes the identified motor units, *MUAP* is the shape of the motor unit action potential, and *ST* is the spike train.

From the spike trains, the motor unit discharge rates and coefficients of variation for the inter-spike interval were estimated. CSTs were generated as the sum of motor unit spike trains recorded from both muscle regions. Individual motor unit spike train comprised a vector where each entry indicated one sample that was assigned the value 1 if an action potential in the corresponding motor unit was identified at the time instant and 0 otherwise. For each contraction, up to 100 random permutations of two CSTs comprising different number of motor unit spike trains were generated. For example, if six motor units were identified from the two intramuscular EMG recordings in a given contraction, all combinations of two CSTs consisting of three motor unit spike trains each were generated. For all permutations, coherence analysis was performed between the two detrended CSTs using the Welch’s averaged periodogram in windows of 3 s, with 50% overlap. The average coherence spectrum of all permutations was extracted for further analysis. The peak values of the coherence in the delta (0–5 Hz), alpha (5–15 Hz), and beta (15–35 Hz) bands were identified. In addition, the average value of the coherence function in the bandwidth 0–35 Hz was calculated. The coherence between the two detrended surface EMG signals was calculated in a similar way as for the CSTs, with and without EMG rectification. In addition, the coherence was calculated for rectified EMG signals that were high-passed filtered (second-order Butterworth) prior to rectification. The cut-off frequency of this filter was 250 Hz as in previous studies (Boonstra and Breakspear, 2012; Laine and Valero-Cuevas, 2017). The level for significant coherence was determined as described by Rosenberg et al. (1989).

The association between coherence values estimated from motor units and from EMGs was tested using linear correlation analysis. The coherence values were transformed into standard Z scores (Rosenberg et al., 1989) prior to this analysis. Here, the CST–CST coherence in the cases with 3 motor units per CST was used (i.e., trials where less than 6 motor unit spike trains were identified were excluded). If both trials performed by the same subject at each contraction level were included, the average values of the EMG–EMG and the CST–CST coherence were used for the analysis. The level of significance was set to 0.05.

## Results

The average MVC torque was 64.9 ± 16.3 Nm (range: 42.7–88.9). Across all trials and subjects, a total of 115 motor unit spike trains were identified at 5% MVC and 150 at 10% MVC from the intramuscular EMG. Motor units identified from the two recording sites were always unique for each site. At 5% MVC, at least 6 motor unit spike trains were identified in 11 out of the 20 trials (from seven subjects), while this number was 14 out of the 20 trials (eight subjects) at 10% MVC. In the included trials, 8.2 ± 1.3 and 9. 6 ± 2.6 motor unit spike trains were identified per trial at 5% and 10% MVC, respectively. The average motor unit discharge rates for the included spike trains were 9.6 ± 1.3 pps at 5% MVC and 9.8 ± 1.7 pps at 10% MVC and the coefficient of variation for the inter-spike interval was 14.2 ± 4.0% (5% MVC) and 13.9 ± 4.9% (10% MVC). Across all subjects, the average SIR, indicating the contribution of the decomposed motor units to the surface EMG signals, was 18.5 ± 11.9% for 5% MVC and 16.6 ± 10.0% for 10% MVC.

**Figure 2** illustrates representative data from one trial (10% MVC) in one subject. **Figure 2A** shows the intramuscular EMG recordings and the identified discharge times of three motor units. In total, the discharge times of 12 motor units were identified from the two recording sites in this trial. The common input to these motor neurons was estimated using coherence between permutations of CSTs with varying number of motor unit spike trains (1–6 motor unit spike trains per CST). For the trial illustrated in **Figure 2C**, the coherence in the delta band, in the upper alpha band, and in the lower beta band increased when more motor units were included in the CSTs. This indicated that the motor neurons received common input in these bands, although this input was revealed only when analyzing the discharge times of more than one pair of motor units, using CSTs (Negro and Farina, 2012). Coherence was also estimated using the rectified and raw surface EMG signals. For this trial, the common input revealed by the CST–CST coherence analysis (**Figure 2C**) was partly reflected in the coherence from the rectified EMG and from the rectified EMG following high-pass filtering (**Figure 2D**). However, these coherence spectra did not reveal peaks at frequencies < 5 Hz, which was the bandwidth with greatest coherence between motor units. The raw EMG–EMG coherence, on the other hand, did not exhibit any peaks in the full 0–35 Hz band.

**FIGURE 3:**
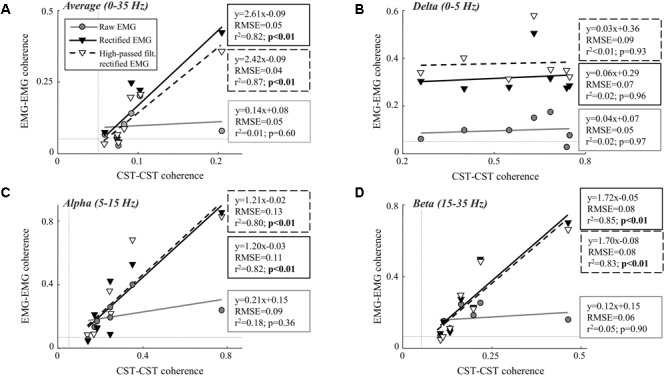
Linear correlation analysis between average coherence (0–35 Hz; **A**), the peak in the delta band (0–5 Hz; **B**), the peak in the alpha band (5–15 Hz; **C**), and the peak in the beta band (15–35 Hz; **D**) calculated between two CSTs and the two surface EMG recordings [raw, rectified, or high-pass filtered (>250 Hz) before rectification]. The contraction level was 5% MVC and CSTs consisting of three motor units each were used. The thin, gray, dashed lines indicate the confidence limits for coherence (0.05; not visible in all panels due to truncation of axes). Note that the figures show raw coherence values, but *p*-values were calculated based on Z-transformed coherence values. Significant correlations (*p* < 0.05) are indicated by bold font.

**FIGURE 4:**
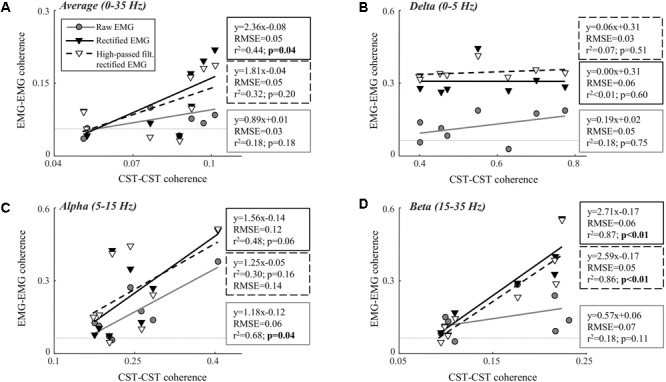
Linear correlation analysis between average coherence (0–35 Hz; **A**), the peak in the delta band (0–5 Hz; **B**), the peak in the alpha band (5–15 Hz; **C**), and the peak in the beta band (15–35 Hz; **D**) calculated between two CSTs and the two surface EMG recordings [raw, rectified, or high-pass filtered (>250 Hz) before rectification]. The contraction level was 10% MVC and CSTs consisting of three motor units each were used. The thin, gray, dashed lines indicate the confidence limits for coherence (0.05; not visible in all panels due to truncation of axes). Note that the figures show raw coherence values, but *p*-values were calculated based on Z-transformed coherence values. Significant correlations (*p* < 0.05) are indicated by bold font.

**Table 1** shows the CST–CST coherence values for each frequency band across the two contraction levels. As in previous studies, the coherence values were highest for the low frequencies (Negro et al., 2016) with little difference across contraction levels (Castronovo et al., 2015). **Figures 3, 4** show the association between these coherence values and those estimated from the surface EMG (raw, rectified, and high-passed filtered prior to rectification). In this way, these figures generalize the observations from the single trial illustrated in **Figure 2**. First, there was no correlation between CST–CST coherence and the EMG–EMG coherence in the delta band at any of the two contraction levels (*r*^2^ ≤ 0.18, *p* ≥ 0.51; **Figures 3B, 4B**). This lack of correlation cannot be explained by a small range of CST–CST coherence values obtained across the trials, since these values were >0.3. Second, there was very little difference between the correlations obtained with rectified EMG and with the EMG that was high-pass filtered prior to rectification. Third, significant correlations were found for the higher frequency bands (>5 Hz), but primarily for the two rectified EMG signals. Specifically, the correlations were statistically significant for alpha and beta bands at 5% MVC, but only for the beta band at 10% MVC (*p* = 0.06 of the alpha band for rectified EMG). The increase in contraction level tended to strengthen the correlation between CST–CST coherence and EMG–EMG coherence from the raw EMG, although this correlation was only significant in the alpha band (*p* = 0.04). Finally, the average CST–CST coherence across all frequency bands was only significantly correlated to EMG–EMG coherence when the EMG was rectified.

**Table 1:** CST–CST coherence (mean ± SD) for all included trials across frequency bands and at 5% and 10% MVC.

	Average (0–35 Hz)	Delta (0–5 Hz)	Alpha (5–15 Hz)	Beta (15–35 Hz)
5% MVC (*n* = 7)	0.10 ± 0.05	0.57 ± 0.18	0.30 ± 0.22	0.20 ± 0.12
10% MVC (*n* = 8)	0.08 ± 0.02	0.55 ± 0.14	0.25 ± 0.08	0.16 ± 0.06

*n indicates the number of subjects included per contraction level.*

## Discussion

We investigated the ability of coherence analysis of the surface EMG to infer common synaptic input to motor neurons. For this purpose, we analyzed the coherence between surface EMG signals recorded from two locations over the tibialis anterior muscle and compared it with estimates of the common input to the motor neuron pool from motor unit data decomposed from intramuscular EMG recordings. Overall, the results showed that the rectified EMG revealed common synaptic input to motor neurons to a greater extent than the raw EMG. While this is in agreement with previous studies (Ward et al., 2013; Dakin et al., 2014), a number of important limitations related to the coherence from the rectified EMG have been identified. First, the association tended to weaken when the contraction intensity increased. While the coherence in the alpha and beta band as well as the average coherence value across the full spectrum were significantly correlated to the CST–CST coherence at 5% MVC, this correlation was significant only for the beta band at 10% MVC. This observation may reflect the effects of amplitude cancellation. As more motor units are recruited, amplitude cancellation increases which has been shown to distort the coherence spectrum (Farina et al., 2013). Conversely, coherence from the raw EMG was only significantly correlated with CST–CST coherence at 10% MVC force (the highest contraction level investigated; Farina et al., 2013). Second, the results showed that input in the delta band was not identified by EMG–EMG coherence unlike common input at higher frequencies. The absence of delta band coherence for the raw EMG analysis was expected since the power of the motor unit action potentials is located at higher frequencies (Negro et al., 2015). However, the bandwidth of the rectified action potentials includes the low frequencies, suggesting that input in the delta band could be detected from the rectified EMG signal. Nonetheless, EMG amplitude cancellation in this frequency band may hinder coherence (Farina et al., 2013; Negro et al., 2015). The results of this study indicate that the effect of cancellation on the rectified EMG coherence is mainly evident at low frequencies. It should be noted, however, that this effect depends on the spectral properties of the motor unit action potentials (Negro et al., 2015) which may differ across muscles and recording systems (Farina et al., 2004). In general, our results indicate that rectification does not necessarily enhance the neural information at all frequency bands with respect to the raw EMG. Third, we found that high-pass filtering the EMG prior to rectification provides no improvement in the association between motor unit and EMG coherence. As previously demonstrated (Boonstra and Breakspear, 2012), this operation tended to increase the beta band coherence slightly (**Figures 3B, 4B**). However, this increase did not improve the ability of EMG–EMG coherence to predict motor unit coherence. Finally, it should be underlined that even in the frequency bands where significant linear relations were found, the root-mean-square errors were high (up to 0.1; **Figures 3, 4**). This suggests that the variability of EMG coherence estimates, with respect to the direct measure of motor unit activity, may be large, which may explain the high cross-session variations in EMG–EMG coherence (Van Asseldonk et al., 2014).

In this study, CSTs were used as the reference for assessing the common input to the motor neuron pool to which the EMG–EMG coherence was compared. Each CST consisted of the spike trains of three motor units. The selected number of motor units for the CST was a compromise between the quality of the data and the quantity of the included trials. By increasing the number of motor units in the CST, a more reliable characterization of the common input is obtained (Farina et al., 2014). The improvement in this characterization achieved by including additional motor unit spike train in the CST depends on the strength of the common input relative to synaptic noise. Although this is likely to vary across muscles and tasks, previous experiments with isometric force matching tasks across several muscles indicate that three motor unit spike trains per CST may be sufficient for adequate characterization of common input (Negro et al., 2016). This is illustrated by the representative data in **Figure 2C** where the substantial peaks in the CST–CST coherence spectrum (<5 and 15–20 Hz) are present with 3 motor units per CST, and the amplitude of these peaks increases steadily with increasing the number of motor units. Whereas it is desirable to maximize the number of motor unit spike trains per CST, the number of motor units that can be reliably decomposed from the intramuscular EMG signals imposes a practical upper limit to this number. In this study, the minimum number of 6 motor units was achieved in 25 out of 40 trials, but if a higher limit were imposed, the number of accepted trials for the analyses would substantially decrease, thereby decreasing the statistical power of the analysis. Nevertheless, it should be emphasized that the CST–CST coherence spectrum is only an estimate of the common synaptic input to the motor neuron pool and that a more conservative interpretation of the results is that the EMG signal does not fully reflect the motor unit behavior. With regard to the degree to which the identified motor units represented the behavior of the entire motor unit pool, the motor unit activity identified in each trial accounted on average for approximately 17% of the surface EMG power (as indicated by SIR). This value may suggest that the identified motor units were not fully representative of the set of units contributing to the surface EMG. However, this is not a severe limitation since the synaptic input can be assumed to be largely common to all recruited motor neurons, as previously shown for contraction levels comparable to those used in the current study (Farina et al., 2014; Negro et al., 2016). Indeed, the input was common to the identified motor neurons as evidenced by the steady increase in CST–CST coherence values as a function of number of motor units per CST (**Figure 2C**). It is unlikely that this behavior was exclusively present in the motor units identified from the intramuscular EMG signals.

The power spectrum of the EMG signal, and thus the EMG–EMG coherence, depends on several factors related to the recording configuration as well as anatomical properties (Lindström and Magnusson, 1977; Farina et al., 2002). This implies that the results of the study do not necessarily generalize to any type of EMG recording. In addition, it is possible that some level of cross-talk was present in the recordings, which would be expected to bias the coherence values toward higher values. Although this risk is inherent in many studies using EMG–EMG coherence in the same muscle (Grosse et al., 2004) or in muscles located close to each other (Farmer et al., 2007; Keenan et al., 2012), it was likely to be minimal in this study due to the relatively long distance between the two surface EMG recording sites and the bi-pennated architecture of the tibialis anterior muscle. Accordingly, the baseline surface EMG coherence values were relatively low (**Figure 2D**). On the other hand, the effect of cross-talk is expected to be more pronounced in fusiform muscles. Instead, the primary limitation of the study was likely the relatively low number of subjects that were included in the analysis. This number was limited to 7 and 8 (for 5% and 10% MVC, respectively) since too few motor unit spike trains were identified in the remaining subjects. Nevertheless, statistical significant correlations were found in several cases. More importantly, these significant cases varied systematically across conditions as theoretically expected. For example (as discussed above), the correlation between CST–CST coherence and rectified EMG–EMG coherence decreased with contraction level.

The most evident disagreement between EMG and motor unit data was related to the strength of coherence in the delta band (**Figures 3, 4**). This was illustrated by the example shown in **Figure 2**, where strong common input in the delta band (**Figure 2C**) was not reflected by any peak in the EMG–EMG coherence (**Figure 2D**). Common synaptic input in the delta band (also referred to as common drive; De Luca and Erim, 1994) is usually related to the voluntary control of force (Farina and Negro, 2015) and accurately reflects the force variability (Negro et al., 2009). Common input to motor neurons in this band is usually strong (Negro et al., 2016), which was also the case in this study as shown in **Table 1**. Nevertheless, it could not be detected from the surface EMG analysis (**Figures 3, 4**). Accordingly, EMG–EMG coherence is usually low in the delta band during steady contractions (Baker et al., 1999; Kilner et al., 1999; Hansen et al., 2002). In contrast, a moderate correlation (peak of cross-correlation function of approximately 0.3) between rectified EMG amplitude and force has been found in steady contractions by intrinsic hand muscles (Yoshitake and Shinohara, 2013). This discrepancy with our results may be explained by a lower level of surface EMG amplitude cancellation in hand muscles with respect to the tibialis anterior because of the smaller number of innervating motor neurons (Feinstein et al., 1955; Farina et al., 2013). Significant coherence in the delta band has also been previously observed in tasks with large force oscillations (Fisher et al., 2012; Moon et al., 2014; Jaiser et al., 2016), and during maintenance of posture (Boonstra et al., 2016), gait (Norton and Gorassini, 2005; Van Asseldonk et al., 2014), or electrical stimulation of the vestibular system (Dakin et al., 2014). These results indicate that EMG–EMG coherence detects delta band common input to motor neurons, only if this is input sufficiently large.

## Conclusion

During low-force static contractions, the estimate of the common synaptic input to motor neurons obtained by the CST analysis is partly associated to the coherence between rectified EMG signals. The results of this study, however, indicate that this association is valid primarily at frequencies > 5 Hz and at low contraction forces (<10% MVC).

## Author Contributions

JD, FN, DeF, SK, NM-K, and DaF contributed to data acquisition and analysis and approved the manuscript for submission and agreed to be accountable for all aspects of the work in ensuring that questions related to the accuracy or integrity of any part of the work are appropriately investigated and resolved. JD, FN, DeF, NM-K, and DaF contributed to the conception of the work, interpretation of data, and drafting and revision of the manuscript.

## Conflict of Interest Statement

The authors declare that the research was conducted in the absence of any commercial or financial relationships that could be construed as a potential conflict of interest.
